# Elder Abuse Victims During the COVID-19 Pandemic: Administrative Data From San Francisco Adult Protective Services

**DOI:** 10.1093/geroni/igab046.282

**Published:** 2021-12-17

**Authors:** Pi-Ju Liu, Aining Wang, Laura Schwab-Reese, Sara Stratton

**Affiliations:** 1 Purdue University, West Lafayette, Indiana, United States; 2 San Francisco Adult Protective Services, Department of Aging and Adult Services, City and County of San Francisco, San Francisco, California, United States

## Abstract

This study examined elder mistreatment victims’ experiences at the beginning of the COVID-19 pandemic. San Francisco Adult Protective Services (APS) caseworkers conducted phone interviews to inquire about clients’ awareness of COVID-19 and unmet needs. Nine-hundred-and-thirty-four (71%) of 1,313 APS’ past clients or their collaterals were interviewed, with 741 (79%) responding positively to COVID-19-awareness questions, and 697 (75%) having no unmet needs. Binary logistic regression with Firth adjusted maximum likelihood estimation method revealed that older persons (p < .05), self-neglectors (p < .05), and victims of neglect (p < .05) were less aware of COVID-19. Unmet needs varied by mistreatment type. Victims of isolation were more likely to have medical needs (p < .05), while victims of emotional abuse were more likely to report loneliness (p < .001). Collaboration between service providers is key in assisting victims experiencing unmet needs to live safely in a public health crisis.

